# Electron Transport in Soft-Crystalline Thin Films of Perylene Diimide Substituted with Swallow-Tail Terminal Alkyl Chains

**DOI:** 10.1021/acs.jpcc.4c06222

**Published:** 2024-12-12

**Authors:** Piotr Ślęczkowski, Yiming Xiao, Jeong Weon Wu, Chihaya Adachi, Lydia Sosa Vargas, David Kreher, Benoît Heinrich, Jean-Charles Ribierre, Fabrice Mathevet

**Affiliations:** † Department of Physics, CNRS-Ewha International Research Center, Ewha Womans University, Seoul 120-750, Republic of Korea; ‡ International Centre for Research on Innovative Bio-based Materials (ICRI-BioM) - International Research Agenda, Lodz University of Technology, Zeromskiego 116, Lodz 90-924, Poland; § Institut Parisien de Chimie Moléculaire, Chimie des Polymères, UMR CNRS 8232, Sorbonne Université, 4 place Jussieu, Paris 75005, France; ∥ Center for Organic Photonics and Electronics Research (OPERA), Kyushu University, Fukuoka 819-0395, Japan; ⊥ Institut Lavoisier de Versailles (ILV), CNRS, Université Paris-Saclay, 45 avenue des Etats-Unis, Versailles F-78035, France; # Institut de Physique et Chimie des Matériaux de Strasbourg (IPCMS), UMR 7504, CNRS, Université de Strasbourg, 23 rue du Loess, Strasbourg 67034, France; ∇ School of Physics and Astronomy, University of St. Andrews, North Haugh, St Andrews KY16 9SS, United Kingdom

## Abstract

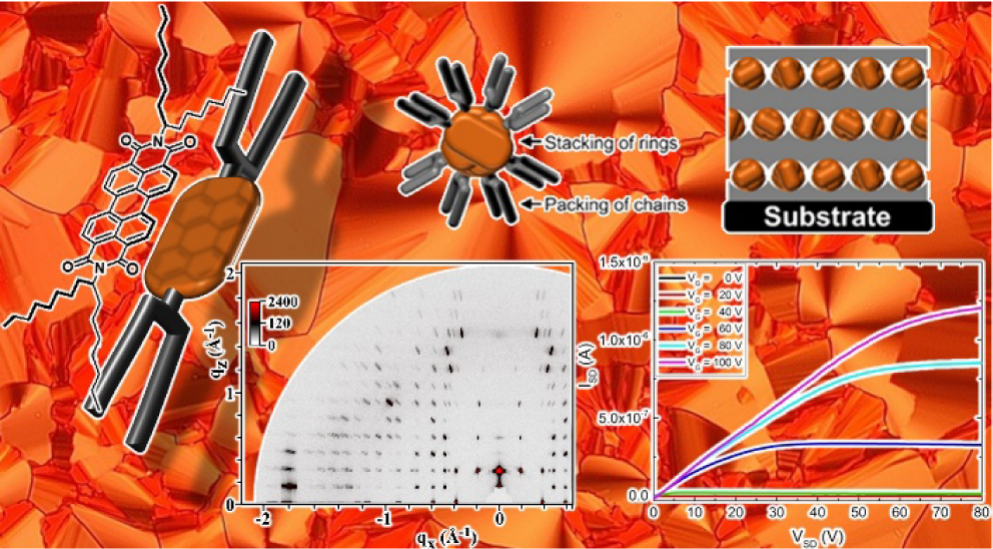

We have examined the structural and electron transport properties of a swallow-tailed *N*,*N*’-bis(1-heptyloctyl)-perylene-3,4:9,10-bis(dicarboximide) (**PDI-C8,7**) in thin films. A comprehensive analysis of material with the use of X-ray scattering methods evidenced the appearance of a new soft-crystalline mesophase that was induced by thermal processing of the swallow-tail PDI derivative. By combining electrical measurements with grazing-incidence wide-angle X-ray scattering (GIWAXS), we show that these morphological changes of thin films boost their charge transport in the organic field-effect transistor (OFET) configuration. The systematic device engineering of OFETs, including device architecture, thermal history, and preparation method of the active layer, resulted in a significant improvement in the electron field-effect mobility and the related performance parameters. In particular, the results demonstrate a strong improvement in the charge transport of **PDI-C8,7** films in their soft-crystalline phase, which originates from the *N*-substitution by swallow-tails. In addition, our study demonstrates that the melt-processing route, a solvent-free and vacuum-free method for the fabrication of organic thin films, represents an efficient strategy for the fabrication of high-performance air-stable *n*-type OFETs.

## 1 Introduction

Perylene diimide (PDI, perylene tetracarboxylic acid diimide) derivatives represent one of the most important and widely studied classes of *n*-type organic semiconducting materials among small organic molecules.^1,2^ As a result of combining high photoluminescence quantum yields and high electron mobility together with excellent photochemical and thermal stability,^3^ PDIs have attracted scientific attention over the past decade.^4^ PDIs are currently available as robust compounds to be applied in organic light-emitting diodes (OLEDs),^5,6^ organic field-effect transistors (OFETs),^7−9^ and organic photovoltaic (OPV) cells.^10,11^ Of special interest remain the solution-processable PDIs, since their development would enable the implementation of inkjet or roll-to-roll fabrication methods, resulting in possible large-area optoelectronic applications.^12^ Besides a few examples of thionation of imide oxygens,^13^ the two most common routes of chemical modifications of PDI comprise: substitution at 1, 6, 7, and/or 12 positions of the PDI core (at the so-called “bay” positions) and *N*,*N*’-substitution (i.e., imidization). While the former can significantly affect the spectroscopic and redox properties (often leading to twisting of the two naphthalene subunits), the latter can be used to tune the solubility, influence the aggregation, and control the molecular packing in the solid state.^1,4^ Extensive studies focused on imide substitutions of PDIs have also evidenced that improved molecular ordering can enhance their functional properties due to an increase of the exciton diffusion length and charge carrier mobility.^14^

One of the effective strategies to obtain high charge carrier mobilities is based on the (bis)imidization of PDI, which generally results in maintaining the planarity of the core and preserving the associated strong π–π interactions.^15^ Previous studies focusing on the *N*,*N*’-substitution showed a dramatic difference between the morphology of PDI self-assemblies depending on the alkyl side-chain topology. The first systematic study of the structure–property relationship for swallow-tail PDIs also provided the comparison of their thermal behavior with respect to PDIs substituted with linear alkyl chains and was reported by Langhals et al.^16^ A particularly significant morphological effect was found for the symmetric PDI compounds containing linear (i.e., *n*-alkyl) and swallow-tail substituents (i.e., having two equally long branches with the branching point close to the PDI core).^17^ While the PDI substituted with a linear dodecyl chain exhibited 1D structures (nanobelts), the steric hindrance provided by the branched nonyldecyl side-chain resulted in the 0D morphology (nanoparticles) for both solution-processing and solvent-vapor-annealing preparation methods. The poor solubility of *n*-alkyl-imidized PDIs in comparison with the swallow-tail PDIs is a result of minimized steric hindrance that promotes the interchain lipophilic interactions. While such interactions are cooperative with the π–π stacking between the aromatic cores, they are strongly reduced by the increased steric hindrance caused by the aliphatic branched side-chains.^18^ Similar behavior was previously reported for *p*-type semiconducting molecules of higher symmetry groups such as hexabenzocoronenes (HBCs).^19^ The low symmetry (*C*_2_) of the PDI core implies a lack of sufficient space filling with terminal chains in the case of *n*-alkyl substituents. Thus, in order to improve solubility, PDIs require either a substitution with more bulky swallow-tails or their exchange to oligooxyethylene substituents, which provide higher conformational flexibility of the C–O bond compared to the C–C bond.^20^ For instance, Funahashi et al. investigated PDIs bearing oligosiloxane chains^21^ and reported that by incorporating pentamethyldisiloxane swallow-tails, it was possible to create liquid crystalline films containing ordered columnar stacks aligned parallel to the substrate, with improved electron mobility up to 0.1 cm^2^ V^–1^ s^–1^.^22,23^

Despite a number of reports on the charge transport properties of swallow-tail PDI derivatives using time-of-flight or space-charge limited current methods in thick films,^21−24^ there is no rigorous study of charge carrier mobility of this class of organic *n*-type semiconductors in OFET configuration. In this context, Guide et al. performed a structure–property-performance investigation of a series of PDIs in the frame of application as the electron-accepting material in OPVs.^10^ The authors have reported the OFET electron mobility of a symmetrical hexylheptyl swallow-tail PDI equal to 4.4 × 10^–5^ cm^2^ V^–1^ s^–1^. However, this work did not contain any further optimization of OFET device performance, e.g., resulting from thermally induced morphological changes of an organic thin film.

**Figure 1:**
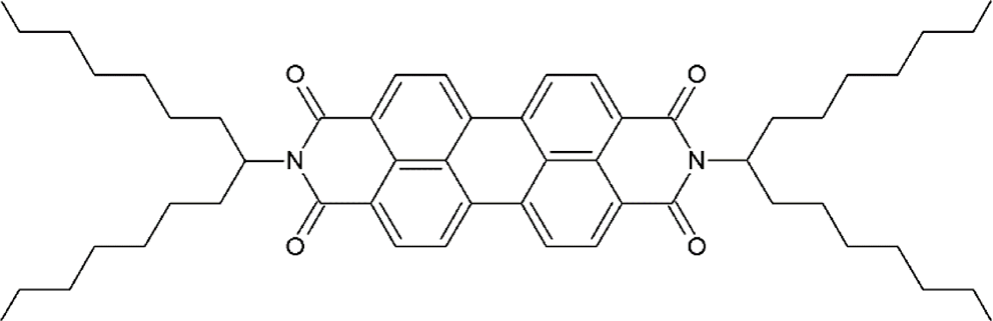
Chemical structure of *N*,*N*’-bis(1-heptyloctyl)-perylene-3,4:9,10-bis(dicarboximide) (**PDI-C8,7**).

In the present work, we have addressed this challenge by a systematic investigation of the charge transport properties in thin films of *N*,*N*’-bis(1-heptyloctyl)-perylene-3,4:9,10-bis(dicarboximide) (**PDI-C8,7**, Figure 1) combined with a comprehensive structural analysis. The morphology and structural properties of the thin films were investigated by X-ray scattering methods, including grazing-incidence wide-angle X-ray scattering (GIWAXS) and small- and wide-angle X-ray scattering (SWAXS). Different types of OFET architectures and thin-film preparation methods (spin-coating, thermal evaporation, solvent-free, and vacuum-free melt-processing) were used to explore the influence of changes in molecular packing and the effects of thermal annealing on the charge transport properties. An increase of the electron mobility by 2 orders of magnitude was achieved in the optimized device architecture and the most favorable molecular packing. This work provides useful insights into the structure–property relationship of perylene diimide substituted with swallow-tail terminal chains and demonstrates the advantages of the melt-processing route for the fabrication of high-efficiency organic optoelectronic devices.

## 2 Results and Discussion

Based on previous density functional theory (DFT) calculation studies showing a torsion angle of 0.0° between the central naphthalene halves of swallow-tail PDI derivatives,^10^ it can be assumed that the polyaromatic core of **PDI-C8,7** molecule is highly planar. In addition, the long swallow-tail alkyl chains substituted at the imide nitrogen atoms make this material highly soluble in common organic solvents. As shown in Figure S1, **PDI-C8,7** has a relatively low isotropization temperature (*T*_i_ = 130 °C) and exhibits a narrow monotropic mesophase on cooling between 110 and 120 °C. Good solubility and the low phase transition temperatures of this material make it suitable for the deposition of thin films using spin-coating and melt-processing^9^ vacuum-free methods. In this context, we prepared sets of samples by means of spin-coating and melt-processing (see the “Experimental Section”) for investigations of the structural and electrical properties of the thin films. Thermal annealing of the spin-coated films was performed to control their molecular packing, optimize their charge transport properties, and establish their structure–property relationship. In addition, due to the mesomorphic properties of **PDI-C8,7**, the melt-processing method could be applied to obtain well-defined polycrystalline organic films.^25^ This melt-processing route takes advantage of the self-healing properties of liquid crystalline materials, allowing to control the film morphology while the material is cooled down from the isotropic phase.^26^ As shown in a previous study, the fine control of the temperature during this improved melt-processing method is the key parameter to control the structural properties of the organic films and improve their charge carrier mobilities.

**Figure 2:**
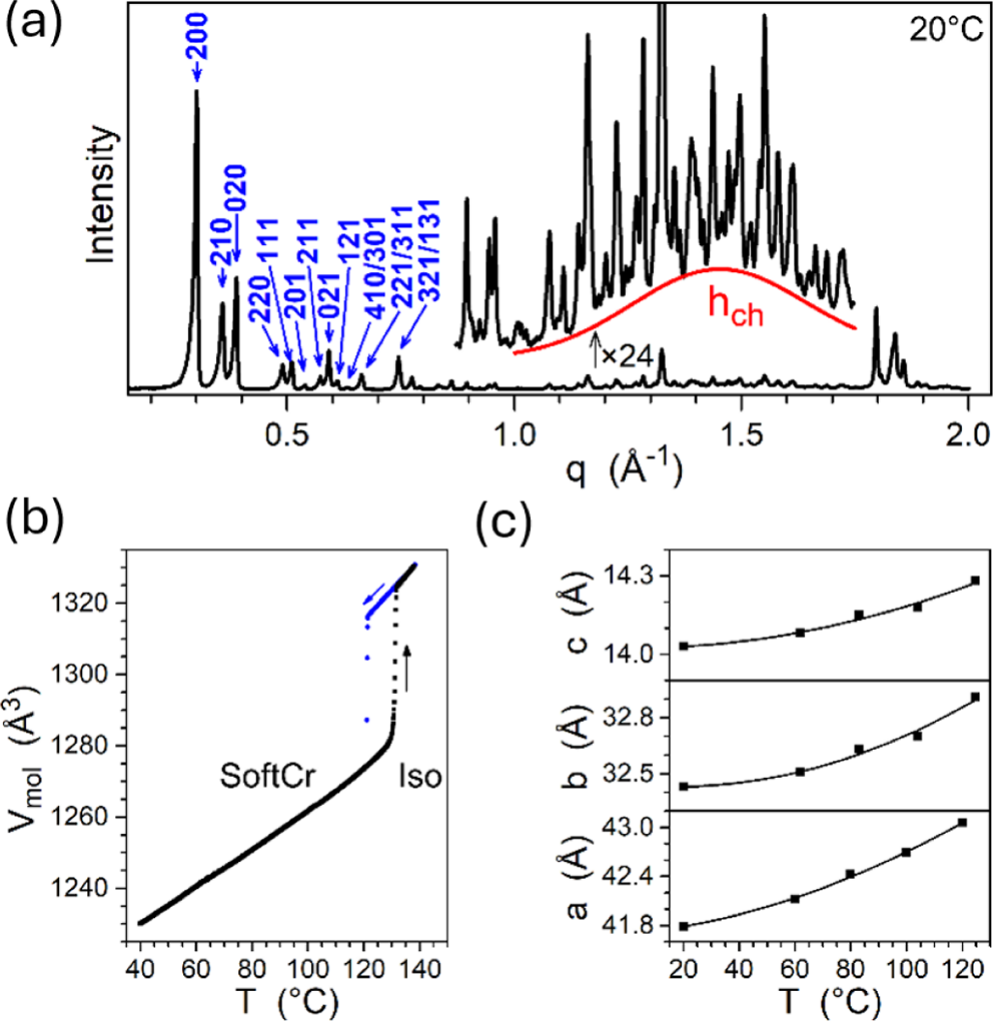
(a) SWAXS pattern of bulk **PDI-C8,7** at room temperature, displaying numerous sharp reflections of an orthorhombic cell (blue labels: Miller indices of the reflections) and a broad scattering signal from molten chains *h*_ch_ (red line); the fully indexed pattern is shown in Figure S3. (b) Molecular volume of **PDI-C8,7** measured versus temperature on crossing the phase transition between a soft-crystal (SoftCr) and isotropic liquid (Iso). (c) Variation of the orthorhombic lattice parameters versus temperature in the SoftCr phase.

To establish the structure–property relationship of the semiconductive **PDI-C8,7** active layers, we performed a detailed analysis of the structural properties of the thin films in conjunction with an in-depth investigation of the bulk structure of the material. A previous work showed that **PDI-C8,7** was found to directly melt from crystal (Cr) to isotropic liquid (Iso) and to form a monotropic hexagonal columnar liquid crystalline phase (Col_hex_) on cooling.^20^ Our characterization of this material by polarized optical microscopy (POM) and differential scanning calorimetry (DSC) is consistent with these previous results (Figure S1). In addition, our DSC measurements evidenced a melting enthalpy of only 20 kJ/mol, which is even lower than 27 kJ/mol of the analogue PDI derivative substituted with linear octyl chains,^27^ and 35 kJ/mol of *n*-pentadecane, which is an *n*-alkane of the same molecular weight as the heptyloctyl chain of **PDI-C8,7**.^28^ This low enthalpy change strongly suggests that the solid state is not truly crystalline, which is in line with the SWAXS results showing a pattern composed of sharp reflections from a long-range correlated 3D structure together with a broad scattering maximum at 1.45 Å^–1^ from the packing of molten alkyl chains (*h*_ch_, Figure 2a). Such a pattern is characteristic of a soft-crystal (SoftCr), defined as a mesophase with a crystal-like 3D arrangement and the presence of molten zones in the cell.^29^ Accordingly, a small molecular volume jump was determined by dilatometry (Figures 2b and S2) since the melting of the 3D lattice does not affect the partial volume of the already molten alkyl chains, unlike crystallized chains whose volume hugely expands on melting.^30^ Specifically, the volume change associated with the melting of two crystallized pentadecyl chains is 171 Å,^3,31^ which is four times larger than the measured volume jump (42 Å^3^). Note that the stacking of PDI in this structure stays essentially rigid, as indicated by solid-state NMR that did not evidence any sign of PDI core motion at the time scale of the experiment.^32^ The combination of SWAXS, dilatometry, and measurements on thin films (see below) allowed us to unambiguously solve the 3D structure, which is orthorhombic with 16 molecules per cell (Figure S3). Note that an orthorhombic geometry was already reported in an early work but with erroneous lattice parameters since the indexation was based on the WAXS data of insufficient quality.^33^ The cell parameters slightly increase with temperature (Figure 2c) as they follow the expansion of the molecular volume (Figure S4 and Table S1).

**Figure 3:**
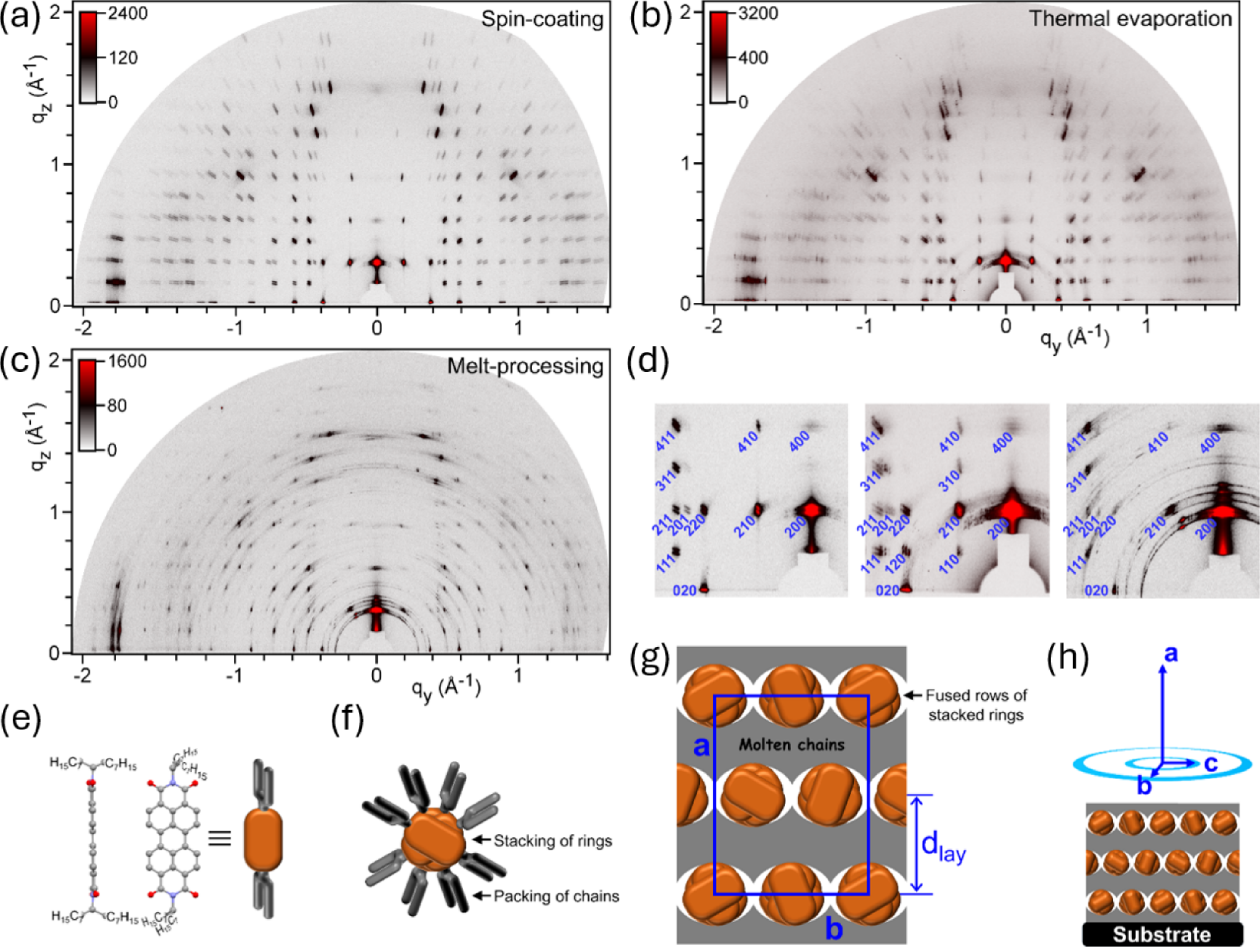
GIWAXS patterns of **PDI-C8,7** thin films deposited by spin-coating (a) or thermal evaporation (b) and annealed at 110 °C, and those of a **PDI-C8,7** thin film prepared by melt-processing (c), which display the reflection spots of oriented domains of the M_orth_ mesophase. (d) Spot indexation of the small-*q* ranges of the patterns from panels (a–c), with the blue labels on the lower left side of spots being the Miller indices of the reflections; the indexation of the full *q*-ranges is shown in Figure S6. (e) Shape and representation of **PDI-C8,7** molecules, as derived from the PDI-C3,2 single-crystal structure CSD-KUZPOL.^37^ (f) Columns of **PDI-C8,7** molecules formed by the rotated stacking of the PDI cores and the packing of the ramified chains in the periphery of stacks. (g) Model of the M_orth_ structure with 16 molecules per cell, *P*2_1_*P*2_1_*P*2_1_ symmetry, and a lamellar substructure of layer periodicity *d*_lay_, based on the side-by-side assembly of PDI columns into layers alternating with molten chains. (h) Morphology of the **PDI-C8,7** thin films characterized by the spontaneous alignment of lamellae parallel to the substrate and the random in-plane orientation of domains.

The thin films of **PDI-C8,7** fabricated by spin-coating, thermal evaporation, and melt-processing were also investigated upon structure and morphology by GIWAXS. The spin-coated and thermally evaporated films showed an undefined structural state when measured directly after deposition (Figure S5). A thermal annealing step at 110 °C rearranged these films into the structure obtained by the melt-processing technique, which was the same as the SoftCr structure of the bulk material (Figures 3 and S6). Whatever the fabrication procedure, the films gave spot patterns of well-oriented SoftCr domains (cf. Figure 3a–d), with the uniform alignment of the *a*-axis on the normal to the substrate and with the random in-plane orientation of the (*b*,*c*)-axes of domains.

To understand the SoftCr self-assembly, additional information about the shape and the interactions of molecules is needed. This could be obtained from model single-crystal structures available in the Cambridge Structural Database (CSD), in particular, those of unsubstituted PDI (PDI: CSD-LENPEZ02^34^) and of PDI *N*-substituted with linear and branched alkyl chains (*n*-octyl (PDI-C8): CSD-GILFUD03;^35^*n*-tridecyl (PDI-C13): CSD-UGUPIX;^36^ ethylpropyl (PDI-C3,2): CSD-KUZPOL^37^). The self-assembly of PDI is ruled by its π-stacking into columns (PDI: stacking distance *h*_π_ = 3.38 Å at 20 °C). For the linear chain derivatives, the substituents are repelled in the periphery of stacked polyaromatic cores by forming a lamellar structure with alternating layers of juxtaposed columns (PDI-C8: *h*_π_ = 3.40 Å at 25 °C) and of crystallized alkyl chains. For PDI-C3,2, the superposition of PDI is hindered by the molecular conformation since the ethyl branches are orthogonal to the aromatic moiety plane, but the stacking is nevertheless maintained (*h*_π_ = 3.43 Å at 23 °C) with an average 40° rotation of facing PDIs (Figure S7 and Table S2). Moreover, the neighboring columns have different orientations to optimize their juxtaposition and the overlaying of the layers. The resulting structure of *P*2_1_*/c* symmetry involves 2 layers, 4 columns, and 8 molecules per cell. **PDI-C8,7** is the heptyloctyl homologue of PDI-C3,2. The introduction of the long swallow-tails results in the formation of aliphatic layers, as for the linear alkyl chain derivatives. However, the repelling of the tails in the adjacent layer requires that they deviate from their initial direction after the branching (Figure 3e). This implies that they keep the conformational disorder of molten chains and incidentally explains the occurrence of a soft-crystal. Moreover, the large overall cross-sectional area of the two molten chain branches imposes an expansion of the PDI layer through lateral tilting of the stacked aromatic rings (Figure 3f and Table S2). The distribution of the branches on top of the surface of columns then induces a helical stacking of PDI with a cell periodicity of four molecules per column. The orthorhombic structure generated by the juxtaposition of the columns and the overlaying of the layers is likely of *P*2_1_*P*2_1_*P*2_1_ symmetry, in agreement with the geometry of the lattice and the systematic extinction of odd (*h*00), (0*k*0), and (00*l*) reflections (model of the M_orth_ structure presented in Figure 3g). One notes the almost cylindrical shape of the PDI columns, which prefigures the formation of the transient Col_hex_ phase on cooling from the isotropic liquid (Figure S1).

It should be mentioned that the PDI orientation inside columns turns by an average of 45° between successive molecules and that this finding is in good agreement with the 30–45° slipped self-assembly suggested by a previous solid-state NMR study.^32^ Also, a corollary of this PDI core slipping is the undulating interface between the layers of juxtaposed PDI columns and those of the aliphatic chains. Tentatively, it is predicted that the lamellar substructure will vanish for some homologue compounds with longer branched chains by generating another structure, among which an orthorhombic columnar self-assembly is a possibility. Regarding the ability of the soft-crystalline structure to transport charges, it can be assumed that the conduction essentially occurs along the columns of stacked perylene rings, i.e., in the *c*-axis direction, while the pathway through juxtaposed columns in the *b*-axis direction should be inefficient, and the one crossing the aliphatic layers in the *a*-axis direction should be insulating. Noticeably, the SoftCr domains of the annealed and melt-processed films spontaneously grew with adequate orientation for OFET devices, namely, with the (*b*,*c*)-plane and therefore with conducting pathways parallel to the substrate plane (Figure 3h). However, the unidimensional charge transport is very sensitive to domain boundaries interrupting the pathways and to domain sizes determining the number of boundaries. The average size of the domains, or its minimum value in the presence of structural disorder, can be evaluated from the GIWAXS patterns. To access the correlation lengths in the plane and normal to the film (ξ_*y*_ and ξ_*z*_), the widths of the reflection spots Δ*q*_*y*_ and Δ*q*_*z*_ were measured from profiles, deconvoluted from the beam shape, and substituted into the Scherrer equation with shape factor *K* = 0.9.^38^ The melt-processed film exhibited dot-like reflection spots of roughly constant width. A set of Δ*q*_*y*_ values collected from 15 spots showed no significant deviation from the beam shape; ξ_*y*_ could thus have any value above the highest measurable one: ξ_*y*_ > 90 nm. A set of Δ*q*_*z*_ values was collected from 11 spots and deconvoluted from multiple scattering,^39^ resulting in ξ_*z*_ = 22 ± 3 nm. The spin-coated and thermally evaporated films exhibited quite narrow spots at low-*q* (the apparent ξ values ranged from 15 to 40 nm) and a gradual broadening with increasing *q*. This feature is the sign of a structural disorder of the second type, i.e., of periodicity fluctuations leading to the loss of the long-range periodicity.^40,41^ The broadening of GIWAXS reflections as large as double the size should be noted for the evaporated films in comparison with the melt-processed samples. Overall, thin films of **PDI-C8,7** revealed the occurrence of a unique soft-crystalline structure, which was also evidenced in the bulk material. The molten zone of alkyl chains constituting the SoftCr mesophase originates from the large stereohindrance of the heptyloctyl swallow-tails. The temperature-driven melt-processing fabrication enabled the largest soft-crystal domain formation, while spin-coated and thermally evaporated thin films revealed higher structural disorder. Even if the increased number of domains/grain boundaries would be detrimental to the charge transport properties, it is shown that thermal annealing of thin films promotes the growth of SoftCr morphology. The appearance of the latter is expected to be a beneficial factor for the charge transport properties of **PDI-C8,7**.

To examine the effects of molecular packing on the charge transport properties in thin films of **PDI-C8,7**, we have fabricated and characterized OFETs of different device geometries, namely, bottom-gate transistors in both bottom-contact (BC) and top-contact (TC) configurations. In the case of the BC-OFETs, the channel length was equal to 20 μm and the channel width was equal to 1 cm. For the TC-OFETs, the channel length was 100 μm and the channel width was either 0.92 or 3.34 cm. Gold was chosen as the material for source and drain electrodes due to its stability. The surface of the SiO_2_ gate dielectric was passivated with a self-assembled monolayer of octadecyltrichlorosilane (OTS), which is a commonly used strategy to avoid trapping of electrons at the semiconductor–dielectric interface by polar silanol groups.^42^

**Figure 4:**
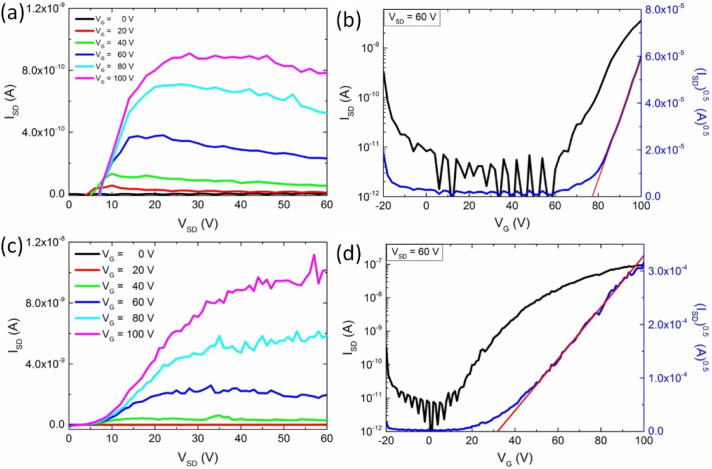
Electrical properties of the bottom-contact OFETs based on spin-coated **PDI-C8,7** films. Panels (a) and (b) show the output and transfer characteristics of the as-prepared samples. Panels (c) and (d) show the output and transfer characteristics of the thermally annealed (110 °C) samples.

Figure 4 shows the representative output and transfer characteristics of spin-coated BC-OFETs. The output characteristics, i.e., source–drain current (*I*_SD_) as a function of the source–drain voltage (*V*_SD_) for a range of gate bias voltages, are displayed in Figure 4a. It is noticeable that they exhibit linear and saturation regimes for positive applied voltages. For all fabricated devices, values of the field-effect mobility (μ) were calculated in the saturation regime, according to a standard procedure reported previously.^43^ They are presented in Table 1, together with the threshold voltage (*V*_th_) and on/off current ratio (*I*_on_/*I*_off_) values. The electron mobility estimated from the transfer characteristics of the as-prepared bottom-contact devices (see Figure 4b) was equal to μ_BC_ = 1.9 × 10^–6^ cm^2^ V^–1^ s^–1^. Moreover, the devices exhibit a moderate on/off current ratio (*I*_on_/*I*_off_) in the range of 10^3^ and high values of the threshold voltage, around 77 V. The behavior of the output characteristics at low *V*_SD_ (i.e., in the 0–7 V range, Figure 4a) together with the large *V*_th_ and the low electron mobility value can be attributed to high contact resistance effects imposed by the interfacial injection barrier.^44^ This is in line with the energy mismatch between the LUMO level of PDI derivatives (−3.8 eV in the case of shorter alkyl swallow-tail PDI^10^) and the Au work-function (−5.1 eV).

**Table 1:** Summary of Data of Field-Effect Mobility, Threshold Voltage, and On/Off Current Ratio of **PDI-C8,7** OFETs Differing in the Method of Preparation of the Active Organic Layer (Spin-Coating, Melt-Processing, Thermal Evaporation), Device Geometry (Bottom/Top-Contact), Conditions of the Measurement, and Thermal History

	Configuration	Measurement conditions	Annealing temperature	Mobility (cm^2^ V^–1^ s^–1^)	Threshold voltage (V)	*I*_on_/*I*_off_
Spin-coated	BC	N_2_	-	1.9 × 10^–6^	77	1.4 × 10^3^
		N_2_	110 °C	7.4 × 10^–6^	32	6.0 × 10^4^
	TC	N_2_	-	3.4 × 10^–5^	57	6.7 × 10^3^
		N_2_	110 °C	4.1 × 10^–4^	38	5.1 × 10^3^
Evaporated	TC	N_2_	110 °C	7.9 × 10^–6^	66	2.9 × 10^2^
Melt-process	BC	ambient	n/a	3.3 × 10^–4^	19	8.6 × 10^5^
		vacuum	n/a	3.1 × 10^–4^	13	4.0 × 10^5^

Since the results of GIWAXS measurements have shown thermally induced morphological changes in **PDI-C8,7** thin films, we then investigated the influence of thermal annealing at 110 °C on the OFET parameters. The output and transfer characteristics of the thermally annealed BC-OFETs are shown in Figure 4c,d, respectively. It is straightforward to notice the change of device performance, manifested by an increase of *I*_SD_ by about 1 order of magnitude (up to ∼1 × 10^–8^ A) in the plateau associated with the saturation regime. Such an increase in *I*_SD_ originates from an improvement in the charge transport properties upon thermal treatment. To gain further insights, analysis of the transfer characteristics of the thermally annealed bottom-gate devices resulted in ^*T*^μ_BC_ = 7.4 × 10^–6^ cm^2^ V^–1^ s^–1^ which corresponds to a 4-fold increase of the electron mobility compared to the value measured prior to the annealing. Another noticeable change is the decrease in *V*_th_, which was equal to 77 V for the as-prepared OFETs and decreased to 32 V in annealed devices. Thermal annealing also strongly influences *I*_on_/*I*_off_, increasing its value by more than 1 order of magnitude, up to ∼10^4^. Overall, the thermal treatment of thin films of **PDI-C8,7** has a positive impact on the electrical performance of the respective BC-OFETs, in agreement with the thermally induced structural transition to the SoftCr mesophase presented above (cf. Figures S5 and 3). It should be noted that the superlinear behavior at low *I*_SD_ is still present in the output characteristics of the devices comprising thermally annealed films, indicating that the contact resistance effects between the Au electrodes and the organic layer were not completely eliminated by the thermal treatment^44^ despite its impact on the molecular packing and the resulting electron field-effect mobility.

**Figure 5:**
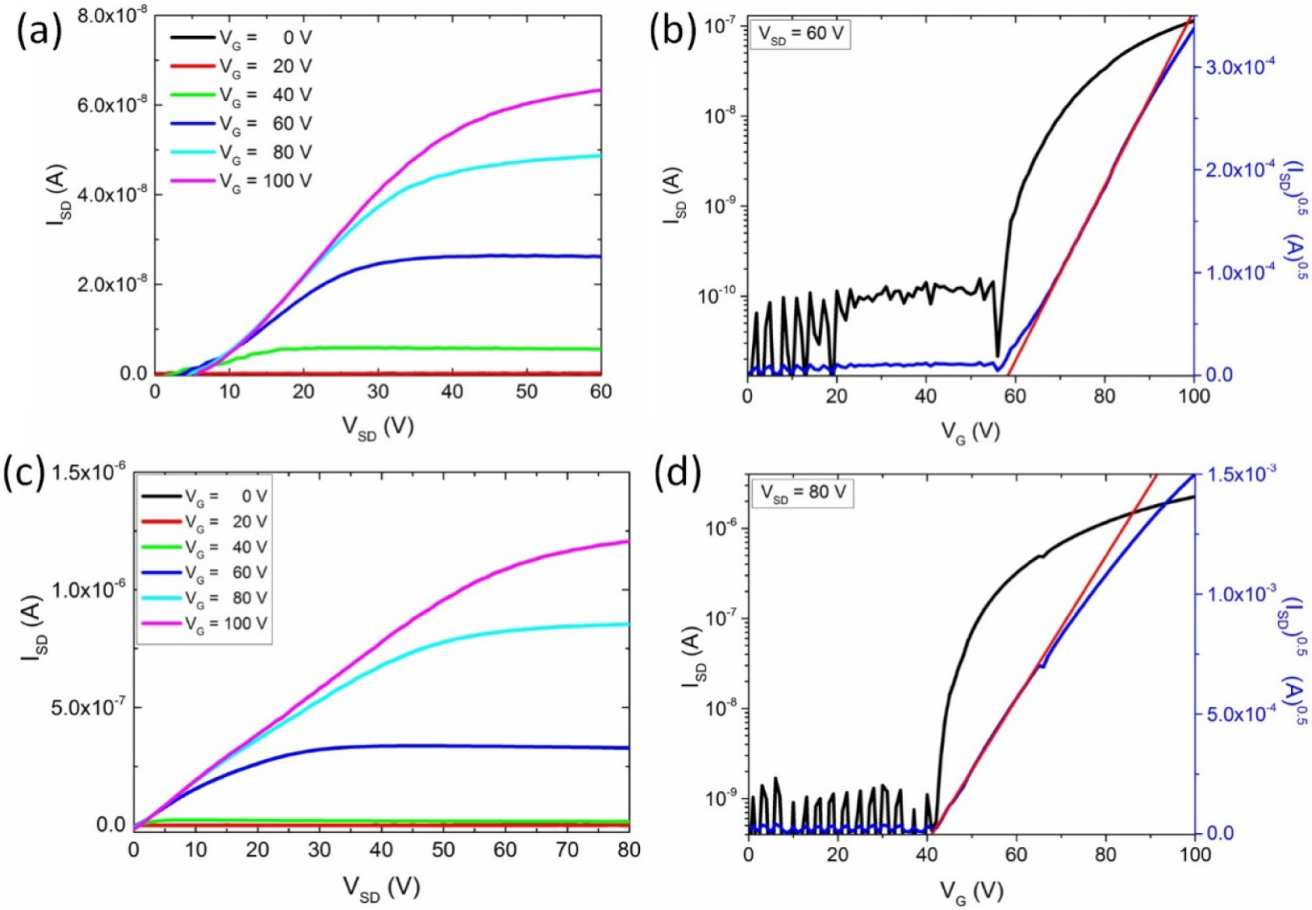
Electrical properties of the top-contact OFETs based on spin-coated **PDI-C8,7** films. Panels (a) and (b) represent the output and transfer characteristics of as-prepared samples. Panels (c) and (d) represent the output and transfer characteristics of the thermally annealed (110 °C) samples.

Since the electrical characteristics of OFETs are often improved in TC configuration due to better charge injection,^42−44^ we have also fabricated TC-OFETs comprising spin-coated **PDI-C8,7** films. Figure 5a shows the output characteristics of the as-prepared top-contact OFETs. While *I*_SD_ in the saturation regime of the TC-OFETs has increased by about 1 order of magnitude compared to the current observed in BC-OFETs (despite their different channel lengths, *L*_BC_ = 20 μm vs *L*_TC_ = 100 μm), the superlinear behavior at low *V*_SD_ is still noticeable, indicating that the contact resistance effects remain unaffected. The electron mobility extracted from the transfer characteristics of the as-prepared TC-OFETs (Figure 5b) is μ_TC_ = 3.4 × 10^–5^ cm^2^ V^–1^ s^–1^, which is higher by more than 1 order of magnitude compared to the value measured in the BC-OFETs. This, in addition to the significant decrease of *V*_th_ down to 20 V, indicates that also for the case of **PDI-C8,7**, the TC geometry is more suitable to achieve better charge transport properties in the OFET configuration.

Considering the beneficial influence of thermal annealing on the BC-OFET device characteristics, we have also investigated the performance of the respective TC-OFETs comprising **PDI-C8,7** thin films, which were thermally annealed at 110 °C prior to the deposition of source and drain electrodes. Subsequent testing of their electrical properties showed, similarly to BC-OFETs, that thermal annealing resulted in the improvement in the electrical performance of the devices. Representative output and transfer characteristics for TC-OFETs are presented in Figure 5c,d, revealing that *I*_SD_ can reach values in the 10^–6^ A range, 2 orders of magnitude larger than those for the as-prepared TC-OFETs. It should also be highlighted that the output characteristics of all top-contact devices containing thermally annealed **PDI-C8,7** films exhibited clear linear and saturation regimes. This is in contrast with the preserved superlinear behavior of *I*_SD_ in thermally annealed BC-OFETs, which evidence an additional positive impact of **PDI-C8,7** active layer thermal annealing on the contact resistance at the organic/metal interface in TC-OFETs.

Consequently, thermal annealing leads to a superior device performance in terms of charge carrier mobilities. The electron mobility of thermally annealed TC-OFETs was ^*T*^μ_TC_ = 4.1 × 10^–4^ cm^2^ V^–1^ s^–1^. This value is almost 2 orders of magnitude higher than those extracted from the BC-OFETs, and it represents the best performance among all the OFETs comprising spin-coated active layers examined in this study. Noticeably, the best electron mobility of **PDI-C8,7** TC-OFETs was found to be 1 magnitude higher than that of the previously reported hexylheptyl swallow-tail PDI analogue.^10^ This occurred even though, in studies to which we refer, top electrodes were made of a lower work-function of Al, potentially facilitating the electron injection to the LUMO level of the active layer material. This also implies that the molecular packing in the SoftCr mesophase has a positive impact on charge transport characteristics in comparison to the crystalline phase of the PDI-C7,6 analogue as evidenced by GIWAXS studies previously.^10^

To gain further insights into the charge transport properties of **PDI-C8,7**, we have then fabricated top-contact OFET devices with **PDI-C8,7** thin films deposited by thermal evaporation. The evaporated films were thermally annealed at 110 °C prior to source–drain electrode deposition. The output and transport characteristics of a representative device are presented in Figures S8a and S8b, respectively. It is straightforward to notice that the *I*_SD_ value of this device remains very low (i.e., maximum *I*_SD_ in the order of 10^–9^ A vs 10^–6^ A in Figure 5c). In contrast to the results obtained in spin-coated annealed TC-OFETs, the superlinear behavior of the output characteristic at low *I*_SD_ indicates a strong contact resistance effect, suggesting a poor interface between the thermally evaporated organic layer and the metallic top-contacts. It was also found that this device exhibited an electron mobility of 7.9 × 10^–6^ cm^2^ V^–1^ s^–1^ in the saturation regime together with a *V*_th_ of 66 V and an *I*_on_/*I*_off_ ratio of 2.9 × 10^2^. Despite having a packing structure similar to that of the annealed spin-coated films, these devices showed much lower performance. The most plausible reason for this result is the severe film dewetting issue that we observed with only the films deposited by thermal evaporation. Overall, these results that are summarized in Table 1 demonstrate a significant impact of the thermal posttreatment on the **PDI-C8,7** thin films and devices. As shown in the GIWAXS analysis, the as-prepared thin films prepared by either spin-coating or thermal evaporation do not show a well-organized molecular packing structure, resulting in poor charge carrier mobilities. Thermal annealing of these films at 110 °C was found to modify their morphology and lead to a unique SoftCr structure with lamellae of stacked aromatic PDI cores aligned parallel to the substrate plane but with a random in-plane orientation. The changes in molecular packing upon annealing are more favorable for efficient charge transport properties in an OFET configuration and can explain the observed enhancement of the electron mobilities in the spin-coated devices. It can be anticipated that further enhancement of the mobility could certainly be achieved by orienting the PDI columns with the use of alignment layers and/or using the mechanical rubbing method.^45^

**Figure 6:**
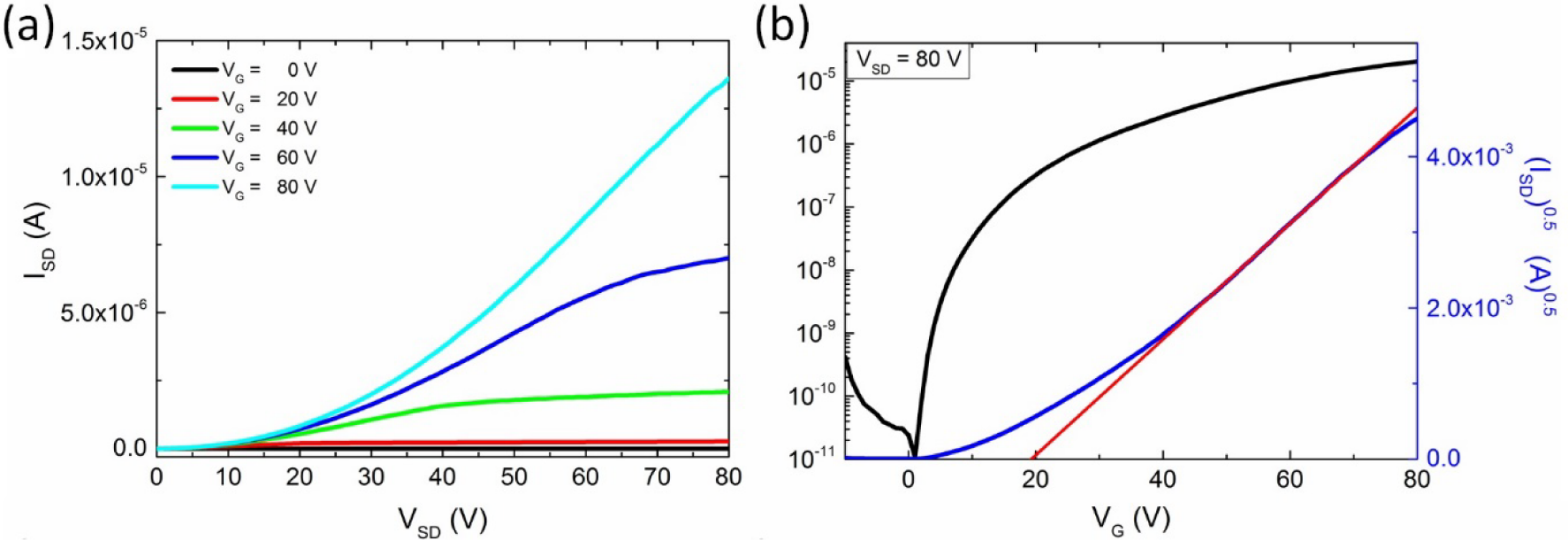
Electrical properties of the bottom-contact OFETs with **PDI-C8,7** films fabricated by the melt-processing route. Panels (a) and (b) represent the output and transfer characteristics of devices characterized in a vacuum.

To complete this study, we have then fabricated BC-OFETs, in which **PDI-C8,7** films were prepared without the use of any organic solvents, by the melt-processing route following the improved method previously reported by Ribierre et al.^46^ Note that melt-processing is not the only method that could have been envisaged to grow large soft-crystalline domains of the present material, especially since the presence of the monotropic columnar phase opens the door to specific, very efficient crystal-growing methods.^47^Figure 6a,b shows the output and transfer characteristics of a representative melt-processed device measured in a vacuum. The melt-processed OFETs showed in the saturation regime an electron mobility μ_mp-vac_ = 3.3 × 10^–4^ cm^2^ V^–1^ s^–1^. The mobility values estimated for the melt-processed OFETs were similar to those of the optimized thermally annealed spin-coated TC-OFETs (i.e., ∼10^–4^ cm^2^ V^–1^ s^–1^). However, it should be highlighted that the OFETs prepared by the melt-processing route exhibited superior characteristics of other device parameters, such as lower *V*_th_ values and *I*_on_/*I*_off_ values larger by 2 orders of magnitude (>10^5^). This clearly demonstrates that melt-processing is an attractive way to prepare electron-transportable organic thin films. In the particular case of **PDI-C8,7**, similarly to the OFETs based on spin-coated and thermally evaporated films, the results of melt-processed OFETs are in agreement with the structural analysis of **PDI-C8,7** films performed by GIWAXS, which evidenced the appearance of the unique soft-crystalline phase. The overall better device performance of the melt-processed devices can be explained by less structural disorder and the larger SoftCr domains with fewer interruptions of conduction pathways by grain boundaries. As was demonstrated, the improvement in the charge transport properties correlates with the size of domains for all implemented fabrication methods. Therefore, promoting the growth of the SoftCr structure is expected to further improve electron mobility in the studied material, less likely to be observed for other swallow-tail PDI derivatives, which exhibit crystalline structure, PDI-C4,3, PDI-C5,4,^48^ PDI-C7,6,^10^ or PDI-C10,9.^17^

## 3 Conclusions

In this work, we investigated the structural and charge transport properties of a symmetric perylene diimide derivative bearing alkyl swallow-tails (**PDI-C8,7**), aiming to establish the respective structure–property relationship in thin films. Our complementary studies evidenced, for the first time, that the incorporation of heptyloctyl swallow-tails at *N*-terminals of PDI plays a crucial role in the origin of a unique soft-crystalline mesophase. By combining detailed GIWAXS measurements with OFET device engineering, the large enhancement of the charge transport properties caused by the thermal treatment was correlated with the appearance of the SoftCr structure in thin films, providing efficient conduction pathways for the OFET configuration. Moreover, it was demonstrated that the presence of swallow-tails readily supports the formation of the SoftCr domains of even larger sizes when the melt-processing technique is used for the fabrication of the **PDI-C8,7** active layer. Overall, this study provides new important insights into the structure–property relationship of PDI derivatives. In particular, the influence of the swallow-tail *N*-substitution on the structural properties as well as on the thin film (post)processing is emphasized, which ultimately enables the use of a more sustainable nonvacuum film deposition, including the most advantageous melt-processing route. This not only paves the way for the development of *n*-type semiconductors, which are lagging behind their *p*-type counterparts, but may also impact the fabrication of high-efficiency organic optoelectronic devices in general.

## 4 Experimental Section

### 4.1 Materials

The material of interest *N*,*N*’-bis(1-heptyloctyl)-perylene-3,4:9,10-bis(dicarboximide) (**PDI-C8,7**) was synthesized according to the literature.^49^ All other chemicals (octadecyltrichlorosilane (OTS) and solvents) were purchased from Sigma-Aldrich and were used without further purification.

### 4.2 POM Experiments

Polarized optical microscopy (POM) was carried out by using a Leica microscope equipped with a Linkam THMS 350 heating plate connected to a Linkam TMS 93 processor.

### 4.3 DSC Experiments

The measurements were carried out with a Q2000 apparatus of TA Instruments, operated at a scanning rate of 10 and 1 °C/min, on heating and cooling.

### 4.4 SWAXS Experiments

The patterns were obtained with transmission Guinier-like geometry. A linear focalized monochromatic Cu Kα1 beam (λ = 1.54056 Å) was obtained by using a sealed-tube generator (600 W) equipped with a bent quartz monochromator. The sample was filled in a sealed cell with an adjustable path. The sample temperature was controlled within ±0.1 °C, and exposure times were equal to 24 h. The patterns were recorded on image plates scanned by an Amersham Typhoon IP with 25 μm resolution (periodicities up to 120 Å). The I(q) profiles were obtained from images by using home-developed software.

### 4.5 Dilatometry Experiments

The measurements were performed with a home-built automatically computer-controlled apparatus, allowing absolute specific volume determination within 0.1% and relative volume variation of 0.01%, for a temperature control within ±0.03 °C.

### 4.6 GIWAXS Experiments

The measurements were conducted at the PLS-II 9A U-SAXS beamline of the Pohang Accelerator Laboratory (PAL) in Korea. The X-rays coming from the in-vacuum undulator (IVU) were monochromated using Si(111) double crystals and focused on the detector using K–B-type mirrors. Patterns were recorded with a 2D CCD detector (Rayonix SX165). The sample-to-detector distance was about 225 mm for an energy of 11.06 keV (1.121 Å).

### 4.7 Fabrication of OFETs

Commercially available chips (Fraunhofer IPMS) were used for the bottom-gate OFETs. Prior to the spin-coating of **PDI-C8,7**, the chips were sonicated in acetone, isopropanol, and chloroform. Then, they were cleaned for 20 min in a UV cleaner.

Top-contact OFETs were fabricated on heavily *n*-doped silicon wafers with a thermally grown 280 nm SiO_2_ layer, purchased from MicroChemicals GmbH. The substrates were sonicated in acetone, isopropanol, and chloroform and then placed in a piranha solution to remove the residues of organic materials and to induce −OH groups at the wafer surface. After rinsing the substrates with deionized water, they were cleaned for 20 min in a UV cleaner and subsequently transferred into a 3 mM OTS/dry hexane solution where they were kept for 90 min. The substrates were finally washed with isopropanol and chloroform in order to get rid of the excess OTS from the surface.

In the case of the top-contact devices, the interdigitated gold source and drain electrodes were evaporated through a shadow mask at a slow rate (0.5 Å/s) on top of either as-prepared or thermally annealed **PDI-C8,7** thin films. To perform thermal annealing, the samples were placed on a hot plate (110 °C) for a period of 60 min in a nitrogen-filled glovebox.

The **PDI-C8,7** films were prepared by spin-coating, thermal evaporation, or melt-processing. For the spin-coated films, the **PDI-C8,7** was dissolved in chloroform at a concentration of 1.2 mg/mL and spin-coated according to the procedure: 3000 rpm for 60 s (in a nitrogen-filled glovebox). For the evaporated samples, the 50-nm-thick **PDI-C8,7** layers were thermally evaporated under a high vacuum (10^–6^ mbar) at a constant rate of 0.5 Å/s. In the case of the BC-OFETs prepared by melt-processing, the channel length and width were 25 μm and 7.6 cm, respectively. The OTS-passivated bottom-contact chips were used, onto which an OTS-treated microscope cover-glass was placed. A small (<0.1 mg) amount of **PDI-C8,7** was placed at the gap between two surfaces which were thermally heated above the isotropization temperature of **PDI-C8,7**. The thin film was formed by melting of the material, which in consequence filled the space between the cover-glass and chip surface by means of the capillary action, and by subsequently cooling down the sample to induce crystallization. When only a few single-crystal germs were maintained, the sample was cooled down at a very slow cooling rate (<0.1 °C min^–1^) to induce the growth of large millimeter-scale single-crystalline monodomains from single-crystal germs until room temperature was reached. Then, the top cover-glass was carefully removed. OFET characteristics were measured using a double-channel source meter unit (Keithley 4200). The measurements in the case of OFETs containing spin-coated and evaporated thin films were performed inside the glovebox, in order to ensure an oxygen-free and water-free atmosphere. The characterizations of OFETs comprising melt-processed films were done either under 10^–6^ mbar vacuum pressure or in an air atmosphere. OFET device parameters (μ, *V*_th_, and *I*_on_/*I*_off_) were calculated as an average of >5 devices.

## Supporting Information Available

DSC traces of **PDI-C8,7** and polarized optical microscopy data evidencing mesomorphism; dilatometry studies of soft-crystal/isotropic liquid phase transitions; SWAXS patterns of bulk and GIWAXS patterns of thin films of **PDI-C8,7**; molecular shape and molecular packing and OFET characteristics (PDF)
