# Supplementary material for: Electron Transport in Soft-Crystalline Thin Films of Perylene Diimide Substituted with Swallow-Tail Terminal Alkyl Chains
Source: J Phys Chem C Nanomater Interfaces. 2024 Dec 12;128(51):21826–35. doi: 10.1021/acs.jpcc.4c06222 (PMC11684020; doi:10.1021/acs.jpcc.4c06222)
Supplement: Supplementary file 1 — jp4c06222_si_002.pdf [file jp4c06222_si_002.pdf]

Supporting information

Electron Transport in Soft-crystalline Thin Films of Perylene Diimide Substituted with Swallow-tail Terminal Alkyl Chains

Piotr Ślęczkowski,[†,‡] *Yiming Xiao,*[†,§] *Jeong Weon Wu,*[†] *Chihaya Adachi,*[⁋] *Lydia Sosa Vargas,*[§] *David Kreher,*[§,ǂ] *Benoît Heinrich,**[ǁ] *Jean-Charles Ribierre**[,±] *and Fabrice Mathevet**[,§,⁋]

[†] Department of Physics, CNRS-Ewha International Research Center, Ewha Womans University, Seoul 120-750, Republic of Korea

[‡] International Centre for Research on Innovative Bio-based Materials (ICRI-BioM) - International Research Agenda, Lodz University of Technology, Zeromskiego 116, 90-924 Lodz, Poland

[§] Institut Parisien de Chimie Moléculaire, Chimie des Polymères, UMR CNRS 8232, Sorbonne Université, 4 place Jussieu, 75005 Paris, France

[⁋] Center for Organic Photonics and Electronics Research (OPERA), Kyushu University, Fukuoka 819-0395, Japan

[ǂ] Institut Lavoisier de Versailles (ILV), CNRS, Université Paris-Saclay, 45 avenue des Etats-Unis, F-78035 Versailles, France

[ǁ] Institut de Physique et Chimie des Matériaux de Strasbourg (IPCMS), UMR 7504, CNRS-Université de Strasbourg, 23 rue du Loess, 67034 Strasbourg, France

[±] School of Physics and Astronomy, University of St. Andrews, North Haugh, St Andrews, KY16 9SS, United Kingdom

Corresponding authors:

* benoit.heinrich@ipcms.unistra.fr

* fabrice.mathevet@sorbonne-university.fr

* jr43@st-andrews.ac.uk

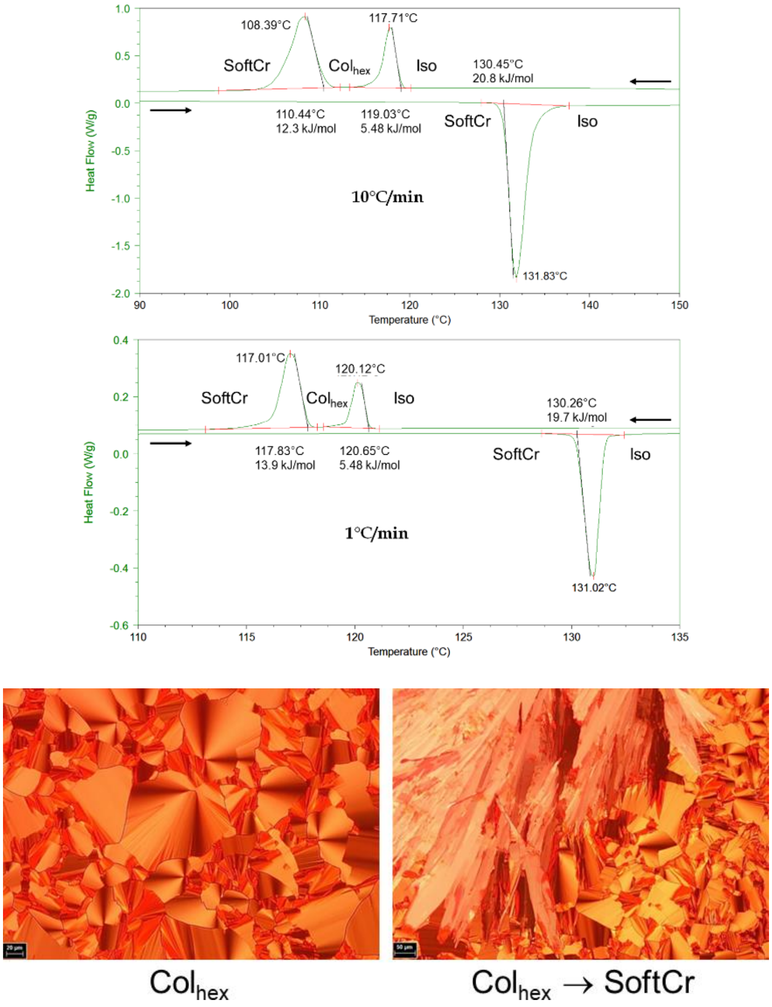

Fig. S1. Top and middle: DSC traces of **PDI-C8,7** (second heating and cooling at 10°C/min and 1°C, endotherm down), showing the transitions between a soft-crystalline solid state (SoftCr), the isotropic liquid (Iso) and a transient, monotropic columnar liquid crystal (Col$_{hex}$). Bottom left: Pseudo focal-conic POM texture characteristic of a columnar liquid crystal observed for the transient phase between 118 and 111°C. Bottom right: transition to soft-crystal phase at 111°C.

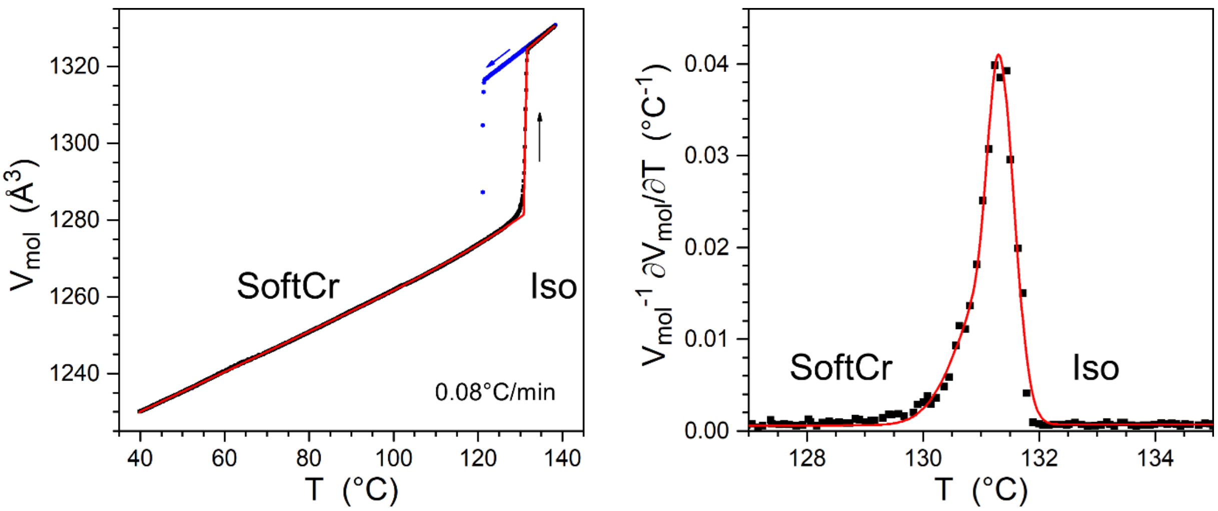

Fig. S2. Left: molecular volume V_{mol} of **PDI-C8,7** versus temperature measured by dilatometry on heating (black squares) and cooling (blue circles) with ramps of 5°C/h and fit (red line). Right: expansion volume coefficient variation versus temperature from dilatometry heating curve derivation. The peak maximum gives a SoftCr → Iso transition temperature of 131.3±0.2°C in agreement with DSC. The volume jump associated to the transition is 42.4 ± 0.4 Å^3 (3.25 ± 0.03%).

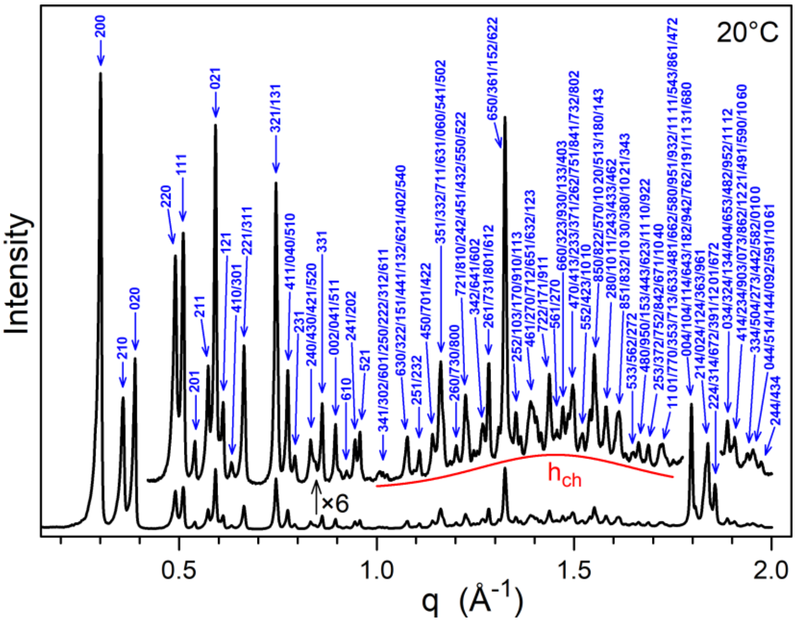

Fig. S3. SWAXS pattern of the soft-crystalline mesophase of bulk **PDI-C8,7** at room temperature, displaying numerous sharp reflections of an orthorhombic lattice (blue labels: Miller indices of the reflections) and a broad scattering signal from molten chains h_{ch} (red line). The pattern was indexed with home-developed software and the lattice parameters are listed in **Table** S1.

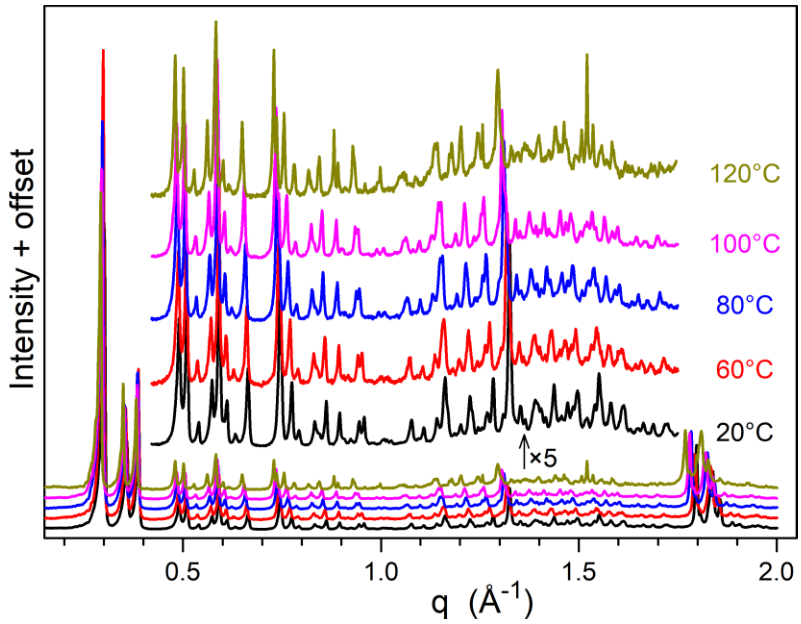

Fig. S4. SWAXS patterns of the soft-crystalline mesophase of bulk **PDI-C8,7** recorded versus temperature on heating. The parameters of the orthorhombic lattice were determined with home-developed software and are listed in **Table** S1.

Table S1. Structural parameters of **PDI-Cn,m** compounds

Compound	T^a	Phase[a]	Structural parameters[b]
**PDI-C8,7**[c]	20	SoftCr	a = 41.79, b = 32.43, c = 14.03, α = β = γ = 90, V = 19014 (Z = 16)
	60		a = 42.12, b = 32.51, c = 14.08, α = β = γ = 90, V = 19280 (Z = 16)
	80		a = 42.42, b = 32.63, c = 14.15, α = β = γ = 90, V = 19586 (Z = 16)
	100		a = 42.69, b = 32.70, c = 14.18, α = β = γ = 90, V = 19795 (Z = 16)
	120		a = 43.06, b = 32.91, c = 14.28, α = β = γ = 90, V = 20236 (Z = 16)
**PDI-C3,2**[d]	23	Cr	a = 34.009, b = 21.248, c = 7.5035, α = β = 90, γ = 100.562, V = 5330.3 (Z = 8)

[a] Temperature (°C) and phase of measurement (SoftCr: Soft-crystalline mesophase with orthorhombic structure; Cr: Crystalline phase); [b] *a*, *b*, *c*, α, β, γ, *V*, *Z*: lattice parameters (Å, °), cell volume (Å^3) and number of molecules per cell; [c]: This work; [d]: Single crystal structure CSD-KUZPOL from the Cambridge Structural Database [SI-1].

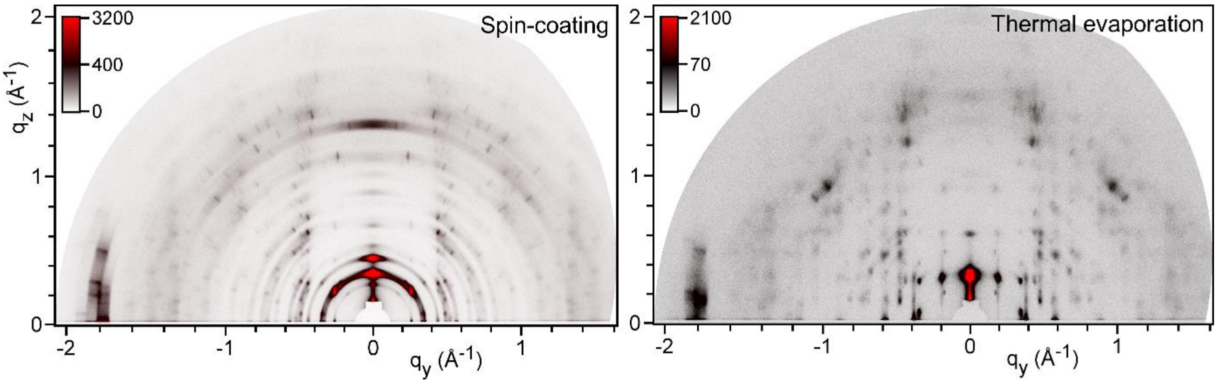

Fig. S5. GIWAXS patterns of **PDI-C8,7** thin films deposited by spin-coating and thermal evaporation without any further thermal treatment. The patterns display a structural state, which is distinct from the annealed films and likely composed of several coexisting crystallographic lattices and preferential domain orientations. The damping and smearing of reflections at high Q demonstrate the high level of structural disorder.

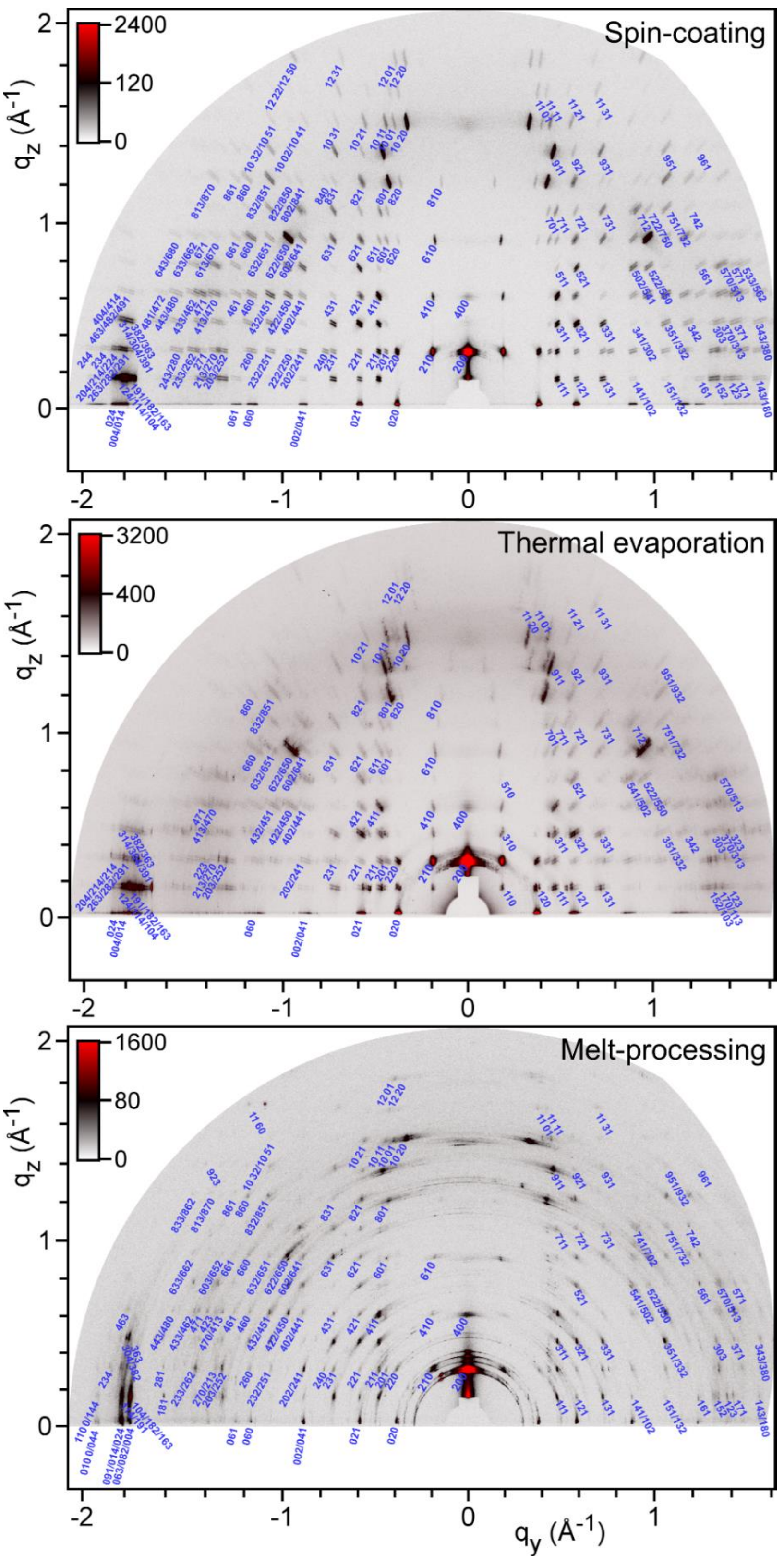

Fig. S6. GIWAXS patterns of **PDI-C8,7** thin films deposited by spin-coating or thermal evaporation and annealed at 110 °C, and of a PDI-C8,7 thin film prepared by melt-processing, which display the reflection spots of oriented orthorhombic domains of the SoftCr mesophase. The blue labels on the down left side of spots are the Miller indices of the reflections.

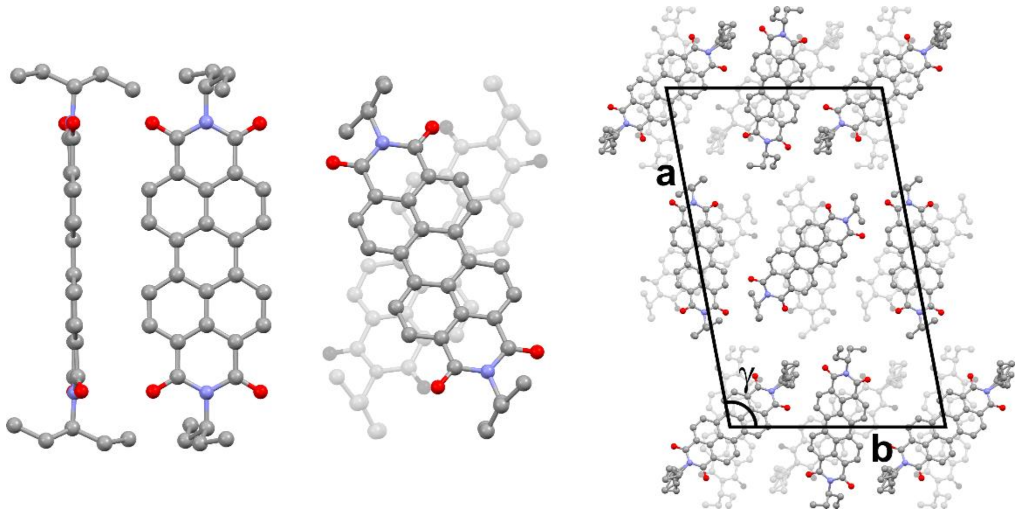

Fig. S7. Views of the molecular shape and the self-assembly of reference **PDI-C3,2** in its single-crystal structure CSD-KUZPOL [SI-1]. Left: The chain branches are directed orthogonally to the PDI plane. Middle: PDI rings π-stacked into columns with rotated in-plane orientations that spread the protruding ethyl tails around the PDI stacks. Right: The columns self-assemble side-by-side with alternating tilt directions into layers, leading to a monoclinic $P2_1/c$ structure with 2 molecules per column and per cell, 2 columns per layer and 2 layers per cell. The lattice and self-assembly parameters are given in **Tables** S1 and S2. The views were generated with the Mercury software; the hydrogen atoms were not displayed for sake of clarity.

Table S2. Self-assembly parameters of **PDI-Cn,m** compounds

Compound	T[a]	Phase[a]	d (n_{lay})[b]	D_{row} (n_{row})[c]	h_{mol} (n_{mol})[d]	h_π[e]	A_{mol}[f]	σ_{PDI}[g]	ψ_{PDI}[h]	σ_{ch}[i]	q_{ch}[j]
**PDI-C8,7**[c]	20	SoftCr	20.90 (2)	16.22 (2)	3.51 (4)	3.43	56.87	30.6	57.5	42.5	1.34
	60		21.06 (2)	16.26 (2)	3.52 (4)		57.22	31.1	57.1	43.7	1.31
	80		21.21 (2)	16.32 (2)	3.54 (4)		57.71	31.4	57.1	44.4	1.30
	100		21.35 (2)	16.35 (2)	3.55 (4)		57.96	31.7	56.9	45.0	1.29
	120		21.53 (2)	16.46 (2)	3.57 (4)		58.74	32.0	57.0	45.7	1.29
**PDI-C3,2**[d]	23	Cr	17.004 (2)	10.624 (2)	3.75 (2)	3.43	39.86	30.6	39.8		

[a] Temperature (°C) and phase of measurement; [b] d, n_{lay}: molecular layer thickness (Å) and number of layers per cell ($d = a/n_{lay}$); [c] D_{row}, n_{row}: spacing of rows of stacked molecules (Å) and number of rows per cell and per layer ($D_{row} = b/n_{row}$); [d] h_{mol}, n_{mol}: spacing of molecules within rows and number of molecules per cell and per row ($h_{mol} = c/n_{mol}$); [e] h_π: π-stacking distance (Å) of PDI rings at temperature T ($h_\pi = h_{mol}\times\cos\psi_{PDI,row}$, where $\psi_{PDI,row} \approx 24°$ is the average tilt angle of PDI rings along rows in the **PDI-C3,2** single crystal structure CSD-KUZPOL [SI-1]); [f] A_{mol}: molecular area i.e. layer area per molecule (Å^2) ($A_{mol} = b\times c/(n_{row}\times n_{mol})$); [g] σ_{PDI}: cross-sectional area (Å^2) of PDI rings at temperature T ($\sigma_{PDI} \approx \sigma_{PDI,T0}\times V_{mol}(T)/V_{mol}(T_0)$, where T_0 is ≈ 20°C, $\sigma_{PDI,T0}$ is the cross-sectional area of PDI rings in the single crystal structure CSD-KUZPOL of **PDI-C3,2** (Å^2) and $V_{mol}(T)/V_{mol}(T_0)$ is the volume expansion coefficient from the dilatometry experiment); [h] ψ_{PDI} is the average tilt angle (°) of PDI rings in the layer plane ($\psi_{PDI} = \arccos(\sigma_{PDI}/A_{mol})$); [i] σ_{ch} is the cross-sectional area of a ramified molten alkyl chain that is equal to twice the area of a linear chain ($\sigma_{ch} = 42.5\ (1 + 7.5 \times 10^{-4}\ (T - 20))$);[SI-2] [j] q_{ch}: chain packing ratio i.e. degree of stretching of chains ($q_{ch} = A_{mol}/\sigma_{ch}$).

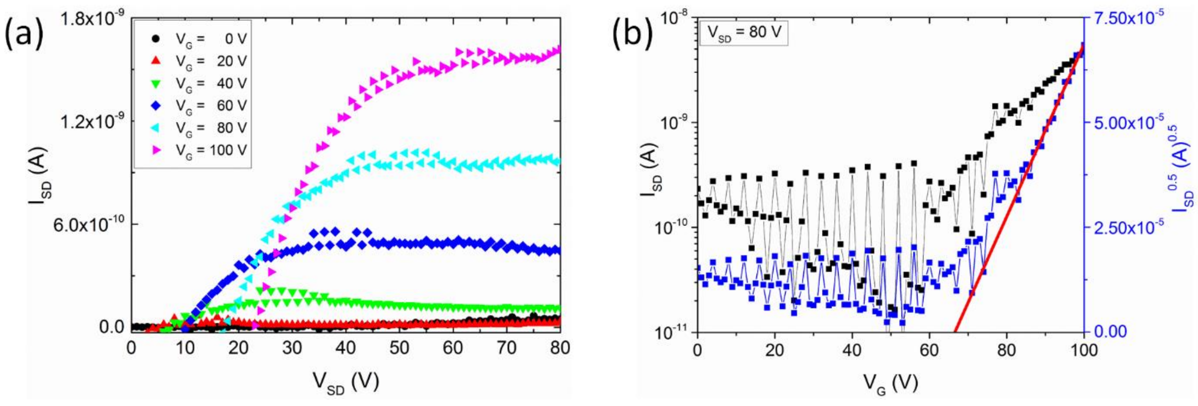

Fig. S8. (a) Output and (b) transfer characteristics of thermally annealed (110°C) top-contact OFETs based on thermally evaporated **PDI-C8,7** films. The surface of the gate dielectric was modified by octadecyltrichlorosilane (OTS) layer.

REFERENCES

[SI-1] Maniukiewicz, W.; Bojarska, J.; Olczak, A.; Dobruchowska, E.; Wiatrowski, M. 2,9-Di-3-pentylanthra[1,9-def:6,5,10-*d'e'f'*]diisoquinoline-1,3,8,10-tetrone. *Acta Cryst.*, **2010**, *E66*, o2570–o2571.

[SI-2] De Gracia Lux, C.; Donnio, B.; Heinrich, B.; Krafft, M.-P. Thermal Behavior and High- and Low-Temperature Phase Structures of Gemini Fluorocarbon/Hydrocarbon Diblocks. *Langmuir*, **2013**, *29*, 5325-5336.
